# A Fixed-Dose Combination, QXOH/Levobupivacaine, Produces Long-Acting Local Anesthesia in Rats Without Additional Toxicity

**DOI:** 10.3389/fphar.2019.00243

**Published:** 2019-03-26

**Authors:** Qinqin Yin, Yujun Zhang, Rong Lv, Deying Gong, Bowen Ke, Jun Yang, Lei Tang, Wensheng Zhang, Tao Zhu

**Affiliations:** Laboratory of Anesthesia and Critical Care Medicine, Department of Anesthesiology, Translational Neuroscience Center, West China Hospital, Sichuan University, Chengdu, China

**Keywords:** QXOH, fixed-dose combination (FDC), preclinical drug development, long-acting local anesthetic, local toxicity, systemic toxicity

## Abstract

QXOH, a QX314 derivative with longer duration and lesser local toxicity, is a novel local anesthetic in preclinical drug development. Previous studies demonstrated that bupivacaine can prolong the effects of QX314. So, we attempted to combine QXOH with levobupivacaine to shorten the onset time and lengthen the duration. In this study, we investigated the efficacy, local and systemic toxicity in rats. In subcutaneous infiltration anesthesia, the inhibition of cutaneous trunci muscle reflex for QXOH-LB was greater than QXOH and levobupivacaine in the first 8 h (QXOH-LB vs. QXOH, *P* = 0.004; QXOH-LB vs. LB, *P* = 0.004). The completely recovery time for QXOH-LB (17.5 ± 2.5 h) was significantly longer than levobupivacaine (9.0 ± 1.3 h, *P* = 0.034) and QXOH (9.8 ± 0.9 h, *P* = 0.049). In sciatic nerve block, QXOH-LB produced a rapid onset time, which was obviously shorter than QXOH. For sensory, the time to recovery for QXOH-LB was 17.3 ± 2.6 h, which was statistically longer than 6.0 ± 1.8 h for QXOH (P = 0.027), and 4 h for levobupivacaine (*P* = 0.001). Meanwhile, the time to motor recovery for QXOH-LB was 7.9 ± 2.8 h, significantly longer than 4 h for levobupivacaine (*P* = 0.003) but similar to 6.0 ± 1.7 h for QXOH (*P* = 0.061). In local toxicity, there was no significant difference of histological score regarding muscle and sciatic nerve in QXOH-LB, QXOH, levobupivacaine and saline (*P* < 0.01). In the combination, the interaction index of LD_50_ was 1.39, indicating antagonistic interaction between QXOH and levobupivacaine in terms of systemic toxicity. In this study, we demonstrated that QXOH-LB produced cutaneous anesthesia which was 2-fold greater than that produced by QXOH or LB alone, and elicited sciatic nerve block with a potency that was 5- and 3-fold that of LB and QXOH, respectively. Local tissue inflammation by QXOH-LB was mild, similar to that induced by LB. This fixed-dose combination led to an antagonistic interaction between QXOH and LB in terms of systemic toxicity. These results suggested that QXOH-LB induced a long-lasting local anesthesia, likely, avoiding clinically important local and systemic toxicities.

## Introduction

Each year, millions of patients elect to have operations or pain management that necessitates the use of local anesthesia (Banerjee et al., 2015). Multimodal analgesia is the mainly contemporary method for pain management which aimed at enhancing pain relief and reducing opioid analgesics requirements by combining with non-steroidal anti-inflammatory drugs (NSAID), local anesthetics and other treatments (Lunn et al., 2015). A short duration is the major deficiency of local anesthetics (Miller et al., 2009). Therefore, prolonging the duration of effect of local anesthetics has been a main purpose of developing new local anesthetics for processes such as local anesthesia, pain management, and chronic pain relief in recent years.

In previous studies, QX314, a lidocaine derivative, produced sensory blockade that was up to six times longer than that of lidocaine (Lim et al., 2007). However, the local and systemic toxicity of QX-314 decrease its potential for clinical use (Barnet et al., 2005; Schwarz et al., 2010; Cheung et al., 2011; Banerjee et al., 2015). In our previous researches, QXOH, a QX314 derivative, showed superior duration of action to that of QX314 and a commonly used long-acting local anesthetic (0.75% bupivacaine) in term of duration with a mild tissue irritation (Invention Patent, China, ZL201410688865.1) (Zhang et al., 2017). Moreover, the published studies have provided useful information that is transient receptor potential cation channel (TRP) activators, such as capsaicin and bupivacaine, can prolong the analgesic effects of QX314 to 24 h (Binshtok et al., 2007, 2009; Ries et al., 2009; Brenneis et al., 2014). Based on this result, we attempted to combine QXOH with levobupivacaine (LB) to shorten the onset time of QXOH and prolong the duration. In a previous study in our laboratory (Zhao et al., 2018), we demonstrated that QXOH at 35 mM with LB at 10 mM (QXOH-LB), was the optimal concentration ratio to achieve the longest duration with mild toxicity. This combination, with a rapid onset, produced an almost 2-fold longer duration of effect than liposomal bupivacaine (Exparel^TM^) did in rats total knee arthroplasty model. Moreover, QXOH-LB was safer than QXOH was in the cardiotoxicity and resuscitation of rats, and the cardiac effects of QXOH and LB in this fixed-dose combination were non-synergistic (Wang et al., 2017). Based on those results, we considered that the fixed-dose combination, QXOH-LB, has a more beneficial clinical application than QXOH.

In our published study, we only determined the efficacy of QXOH/LB in acute post-operation pain animal model. In clinical practice, local anesthetics were often used in surgical anesthesia and chronic pain control. As an important part of preclinical drug development, QXOH/LB should be verified in different local anesthesia animal models. Therefore, in this study, we aimed to determine whether QXOH/LB produces a longer local anesthetic effect in the sciatic nerve block model and infiltration anesthesia models. In addition, we evaluated the local tissue and systemic toxicity. The final goal of this research was to create a novel long-acting local anesthetic.

## Materials and Methods

### Drugs

QXOH was synthesized by Chengdu Institute of Organic Chemistry, Chinese Academy of Sciences (Chengdu, China) according to the method described in patent (Invention Patent, China, ZL201410688865.1). Levobupivacaine hydrochlorides were obtained from Human-well Pharmaceutical Ltd., (Hubei, China). QXOH was diluted with Ultrapure water (Merck Millipore, Darmstadt, Germany). The combination of QXOH and levobupivacaine mixture was prepared by diluting QXOH powder into levobupivacaine solution.

The fixed-dose combination of QXOH at 35 mM with levobupivacaine at 10 mM (pH = 6.49), QXOH 35 mM (pH = 6.38), levobupivacaine 15 mM (equals 0.5%, the commonly used concentration in clinical practice, pH = 5.35) and sodium chloride 0.9% were abbreviated as the terms of QXOH-LB, QXOH, LB and Saline, respectively.

### Animals

Young adult Sprague-Dawley rats (Chengdu Dossy Biological Technology Co., Ltd., China) weighted 250 to 300 *g* were housed at 25°C with free access to food and water under 12 h light/ 12 h dark cycle (lights on at 7:00 AM). All animals were acclimated to experimental environments and experimenters, handled daily to minimize stress-induced analgesia, and randomized into groups. The total number of rats used in the study was 214. Then rats in subcutaneous infiltration anesthesia model and sciatic nerve block model were 8 male rats in each group. To determine of half lethal dose, 150 rats with half males and half females were randomized into 3 groups, and then 10 rats were used for each dose. Experiments involved in animal procedures were strictly conducted in accordance with the Guide for the Care and Use of Laboratory Animals by the United States National Institutes of Health (NIH Publications No. 80–23, revised 1996), and were supervised by the institutional animal experiment ethics committee of Sichuan University (Chengdu, China, Ethical Approval Number, 2015014A).

### Subcutaneous Infiltration Anesthesia Model

Subcutaneous infiltration was performed by subcutaneous injection of 0.1 ml of test solution on the left dorsal region with 27-gage, 1/2-inch hypodermic needles resulting in a 5–8 mm skin wheal which was marked with ink. A similar ink-mark was made on the contralateral side. Six standardized pinpricks were applied at different sites within the wheal on each side by one blunt 18-gage needle attached to a 26 *g* von Frey filament, in order to standardize the force of pinprick (Zhang et al., 2017). Pinprick stimulation elicits cutaneous trunci muscle reflex (CTMR), which is skin movement over the back. An effective subcutaneous infiltration anesthesia was considered when half (3/6) or more CTMRs were inhibited.

### Sciatic Nerve Block Model

Anesthetized with 1–2% isoflurane in oxygen *via* a transparent custom-made plastic mask, the rat was placed on a thermal blanket. A 29-gage needle was vertically inserted at the one-third of the line connecting the greater trochanter and ischial tuberosity (caudal to the greater trochanter) (Yin et al., 2016). 0.2 ml of test solution was deposited once the tip of the needle encountered the ischium (Sagie and Kohane, 2010; Yin et al., 2016). Then the animal was transferred to a transparent plastic chamber for observation.

Neurobehavioral measurements were summarized as previously described (Sagie and Kohane, 2010; Yin et al., 2017). In hot plate test for nociceptive block assessment, a rat was vertically hold in order to make the paw of the injected side placed on a heated metal plate (the temperature of the plate was 56°C, RB-200 Hot Plate, Chengdu Techman Software Co., Ltd.,). The time for the rat to remove the paw after sensation of noxious heat is the paw withdrawal latency (PWL). A cutoff time was set to 12 s to avoid skin damage. In extensor postural thrust test (EPT) for motor blockade measurement, a rat was held vertically to make the hind paw placed against an electronic balance. The force exerted by the hind limb to stand upright was measured in grams (Thalhammer et al., 1995). Baseline values of the above neurobehavioral tests were obtained before the experiments. Neurobehavioral tests were performed at the following time points after perineural injection: 10 min, 30 min, then hourly within 4 h, then every 2 h within 8 h, 12 h, and then every 12 h thereafter until test values returned to baselines. The neurobehavioral tests were performed in a blinded fashion, observers were blinded to the treatment each rat received. At each time points, three repeated measurements of EPT force were taken and averaged; the PWL assessment was taken only once. PWL < 7 s and muscle strength recovered under 60 *g* were considered as nociceptive and motor blockade, respectively (Templin et al., 2015).

### Histological Analysis of Local Tissue Toxicity

Rats were euthanized by overdose of peritoneal pentobarbital at the fourteenth day after sciatic injections. The sciatic nerve with adjacent tissues were harvested and immersed in 4% formalin, then the tissues were stained with Hematoxylin-Eosin (Yin et al., 2016) and Luxol Fast Blue (Zhang and Chen, 2013). Evaluation of tissue morphological changes was performed by three pathologists in a blinded fashion with BX51 microscope system (Olympus, Tokyo, Japan) (Shackelford et al., 2002). In Hematoxylin-Eosin, inflammation was scored according to a previously established scoring standard (Padera et al., 2006). A 0–4 score system was used to measure the degree of local toxicity in sciatic nerve and adjacent muscles. Meanwhile, we evaluated the local tissue injury by necrosis, degeneration and vacuolation in sciatic nerve and muscles. Due to peripheral nerve seldom regenerates, morphological changes such as demyelination, nerve fiber vacuolation and necrosis often indicate irreversible nerve damage. The 0–4 score system was 0 = normal; 1 = 0–25% of area involved; 2 = 25–50% of area involved; 3 = 50–75% of area involved; and 4 = 75–100% of area involved (Shackelford et al., 2002).

### Determination of Half Lethal Dose (LD_50_)

To investigate systemic toxicities of QXOH, LB and QXOH-LB, a single intravenous injection model was used to mimic a rapid vascular absorption. Half lethal dose (LD_50_) was calculated using maximum likelihood method for toxicity evaluation and comparison (Templin et al., 2015; Wang et al., 2017). According to the result of preliminary experiment, we chose the dose level for QXOH-LB was 10, 13.3, 16.7, 20 and 25 mg/Kg (Refer to the dose of QXOH in combination); for QXOH was 9, 13.5, 18, 27 and 36 mg/Kg; for LB was 4.8, 7.2, 9.6, 12 and 14.4 mg/Kg. Test solutions were injected *via* tail vein with a total volume less than 2 mL and an injection speed of 0.25 mL/s. The half lethal dose experiment of each drug was performed only once. Furthermore, we determined the interaction between QXOH and LB in systemic toxicity using the interaction index (γ) of Isobolographic analysis (Gessner, 1995; Tallarida, 2002). The interaction index was calculated as the following equation:

$$\frac{a}{A}+\frac{b}{B}=\gamma$$

Where *A* and *B* represent the LD_50_ for QXOH and LB alone, respectively, a and b are the LD_50_ for each drug in combination. γ = 1, indicating additive interaction; γ < 1, indicating synergistic; and γ > 1 is antagonistic.

### Statistical Analysis

All experiments were a randomized, blinded and controlled experimental design. All statistical analysis was calculated using SPSS (version 23, SPSS, Chicago, IL, United States). Normal distributed data were presented as mean ± SEM, difference among groups was detected by one-way *ANOVA*, and multiple comparison was conducted by *Dunnett* test. Some of sciatic nerve block data are skew-distributed, the comparison among groups was performed by *Kruskal-Wallis* test. LD_50_ with 95% confidence interval were calculated using *Probit* analysis and maximum likelihood method (Templin et al., 2015). Significant difference between the examined groups was considered when *P* value less than 0.05 (two-sided).

## Results

### Subcutaneous Infiltration Anesthesia Model

**FIGURE 1:**
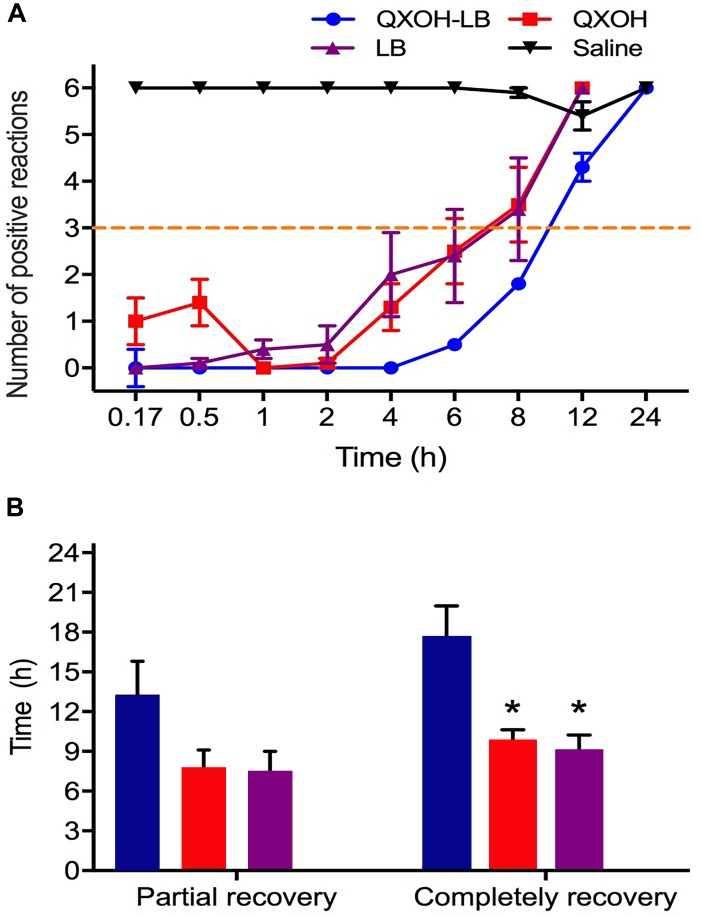
The potency **(A)** and duration **(B)** of subcutaneous anesthesia with QXOH-LB (QXOH at 35mM with levobupivacaine at 10 mM), QXOH 35mM, LB 15 mM, and Saline in rats (*n* = 8 in each group). Subcutaneous infiltration was performed by subcutaneous injection of 0.1 mL of test solution. Data are expressed as mean ± SEM. ^∗^*P* < 0.05. (In **B** QXOH-LB, QXOH and LB were labeled by blue, red, and purple, respectively).

The onset time of subcutaneous infiltration anesthesia for QXOH was 25.0 ± 8.2 min, compared with 10.0 ± 0 min for the other treated groups (LB and QXOH-LB, *P* = 0.113). For the first 8 h (Figure 1A), the inhibition of CTMR by QXOH-LB was greater than that by QXOH alone and LB alone. (QXOH-LB vs. QXOH, *P* = 0.004; QXOH-LB vs. LB, *P* = 0.004). There was no statistical difference between partial recovery time among QXOH (7.8 ± 1.3 h), LB (7.5 ± 1.5 h), and QXOH-LB (13.3 ± 2.5 h, *P* = 0.216, Figure 1B). However, the completely recovery time for QXOH-LB (17.5 ± 2.5 h) was significantly longer than that for LB (9.0 ± 1.3 h, *P* = 0.034) and QXOH (9.8 ± 0.9 h, *P* = 0.049, Figure 1B).

### Sciatic Nerve Block Model

**FIGURE 2:**
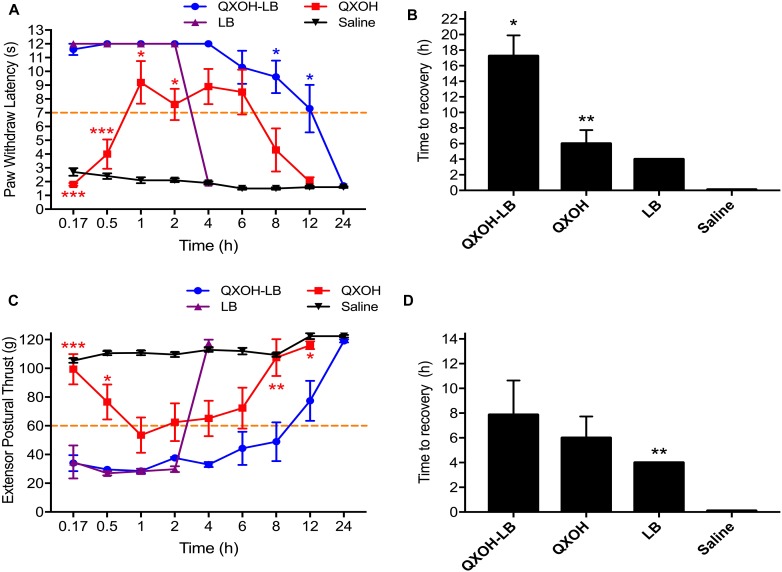
The actual measurement value and recovery time of sensory **(A,C)** and motor **(B,D)** function in sciatic nerve blockade with QXOH-LB (35 mM QXOH with 10 mM levobupivacaine), QXOH 35 mM, LB 15 mM, and Saline in rats (*n* = 8 in each group). 0.2 ml of test solution was used in sciatic nerve block model. Data are expressed as mean ± SEM. ^∗^*P* < 0.05, ^∗∗^*P* < 0.01, and ^∗∗∗^*P* < 0.001.

All the rats injected with saline showed no difference from baseline. For sensory function (Figure 2A), QXOH-LB and LB both induced a rapid onset of nociceptive block (10.0 ± 0 min), which was obviously shorter than that of QXOH was (50.0 ± 6.3 min, *P* = 0.004). The time to recovery for QXOH-LB was 17.3 ± 2.6 h, which was statistically significantly longer than the 6.0 ± 1.8 h and 4.0 ± 0 h for QXOH (*P* = 0.027), and LB (*P* = 0.001), respectively (Figure 2B).

Complete muscle paralysis developed rapidly following QXOH-LB and LB administration, whereas it was slow and incomplete following QXOH (Figure 2C). The onset time of motor block for QXOH was 46.7 ± 16.1 min, which was longer than that for QXOH-LB and LB (12.5 ± 2.5 min and 12.5 ± 5.4 min, respectively, *P* = 0.011). The time to motor recovery for QXOH-LB was 7.9 ± 2.8 h, which was significantly longer than 4.0 ± 0 h for LB (*P* = 0.003) but similar to 6.0 ± 1.7 h for QXOH (*P* = 0.061, Figure 2D).

### Sciatic Nerve and Muscle Tissue Toxicity

**FIGURE 3:**
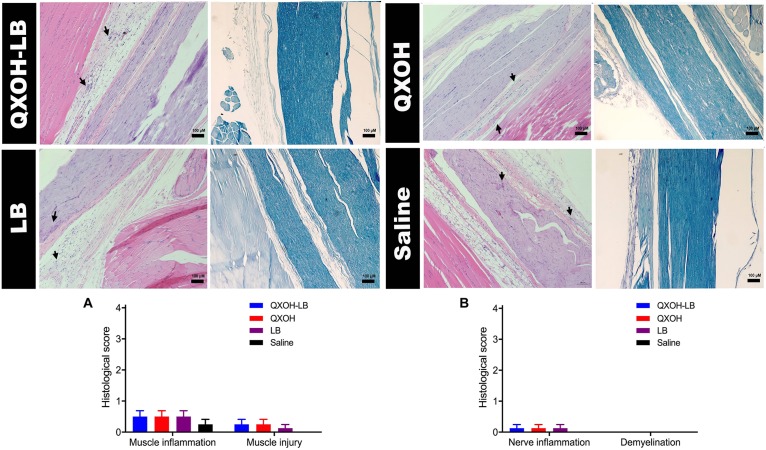
Representative images of Hematoxylin-Eosin and Luxol fast blue stained sections (*n* = 8 in each group), and the histological scores of muscle **(A)** and nerve tissues **(B)**. Data are expressed as mean ± SEM. QXOH-LB: 35 mM QXOH with 10 mM levobupivacaine; LB: levobupivacaine.

Fourteen days after the injections, rats were euthanized for histological analysis, and the results are presented in Figure 3. No pathological morphological changes were observed in the muscle and sciatic nerves of rats treated with saline. There was no significant difference in scores of the muscle and sciatic nerve analyses in the QXOH-LB, QXOH, LB, and saline groups (*P* < 0.01). Histological examination revealed mild tissue inflammation in the QXOH-LB-treated group, with muscle inflammation scores between 0 and 1, similar to the 0.5% LB-treated group (*P* < 0.001, Figure 3A). Muscle injury, including degeneration and granuloma formation was no significant difference (*P* < 0.01) among QXOH, LB and QXOH-LB. Due to peripheral nerve tissue seldom regenerates, morphological changes within the epineurium such as demyelination, necrosis and vacuolation often indicating irreversible nerve damages (Padera et al., 2006). In this study, none of these conditions were observed. The histological results suggested the local tissue reactions induced by QXOH-LB were benign.

### Systemic Toxicity

After single regional injection of local anesthetics, the drugs are absorbed systemically by circulation, which might cause central nervous system toxicity, cardiotoxicity and respiratory function damage, and even lead to death. We attempted to simulate an accidental intravenous (I.V.) administration to evaluate the systemic toxicity in preclinical experiments. In addition, we evaluated the interaction between QXOH and LB at the fixed-dose combination.

**FIGURE 4:**
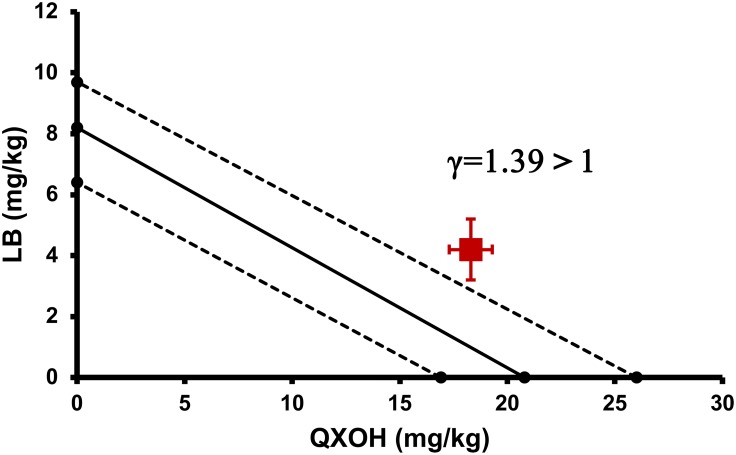
Isobolographic analysis between QXOH and LB for half lethal dose.

The LD_50_ of QXOH alone was 20.8 (95% confidence interval [CI]: 16.0–26.0) mg⋅kg^-1^ compared with 18.3 (95% CI: 15.9–20.9) mg⋅kg^-1^ for QXOH in combination with LB. The LD_50_ of LB alone was 8.2 (95% CI: 6.4–9.7) mg⋅kg^-1^, compared with 4.2 (95% CI: 3.7–4.8) mg⋅kg^-1^ for LB in combination with QXOH. Although the LD_50_ of QXOH-LB was lower than each agent used alone. However, the interaction index (γ) of the LD_50_ by the isobolographic analysis was 1.39, greater than 1, which was indicated an antagonism effect in combination. As the Figure 4 illustrated, the red spot, which indicated the LD_50_ of QXOH and LB when used in the fixed-dose combination, located above the line connecting the LD_50_ of each drug when used alone.

The results demonstrated that QXOH-LB produced cutaneous anesthesia, which was 2-fold longer than that produced by either QXOH or LB, and the combination-elicited sciatic nerve block was 5- and 3-fold that induced by LB and QXOH, respectively. The local tissue inflammation induced by QXOH and QXOH-LB was mild, similar to that by LB. The I.V. toxic doses for QXOH-LB were several-fold of those applied locally. The fixed-dose combination showed an antagonistic interaction between QXOH and LB in terms of systemic toxicity.

## Discussion

Long-lasting local anesthesia is an ideal approach for post-operative pain management. Strategies that have been used to achieve this goal include local anesthetics with additives (Candido et al., 2002; Movafegh et al., 2006; Brummett et al., 2008, 2009, 2011), sustain-release materials (Lambrechts et al., 2013; Yin et al., 2016), sodium channel blockers such as tetrodotoxin and antidepressant, new chemical molecules (Banerjee et al., 2015; Templin et al., 2015) and co-application with chemical permeation enhancers (Sagie and Kohane, 2010) or synergists (Shankarappa et al., 2012). However, to the best of our knowledge, the ideal long-lasting local anesthetics are not clinically available. The obtained results suggested that QXOH-LB induced a long-lasting local anesthesia, likely, avoiding clinically important local and systemic toxicities.

The liposome-loaded bupivacaine known as Exparel^TM^ is recommended by the U.S. Food and Drug Administration (FDA) for skin infiltration anesthesia but not yet for nerve blockade (Lambrechts et al., 2013). A previous study reported that Exparel^TM^ produced sciatic nerve blockade for 4 h in rats, with peri-neural inflammation greater than that induced by 0.5% bupivacaine (McAlvin et al., 2014). Using a knee surgery rodent model, we reported that QXOH-LB produced effective analgesia for 9.8 h and complete nerve block for 7 h, compared with 4.8 h and 2 h for Exparel^TM^, respectively (Zhao et al., 2018). Other published study developed a novel mimetic of QX314, namely EN3427, which produced effective and long-lasting subcutaneous infiltration anesthesia and sciatic nerve block in rats. However, peri-sciatic nerve injection of 1 mg EN3427 caused localized myopathy and neuropathy, leaving local tissue reactions open to debate (Banerjee et al., 2015). The recently reported site-1 sodium-channel blocker, neosaxitoxin (neoSTX), also a member of a broad group of natural neurotoxic alkaloids, has shown potential efficacy for long-lasting local anesthesia. NeoSTX at 3 μg⋅kg^-1^ with 0.2% bupivacaine (neoSTX-Bup) produced rat sciatic nerve blockade for 6 h, and adding epinephrine into this combination further prolonged the block to 48 h (Templin et al., 2015). However, neoSTX at dosed greater than 3 μ⋅kg^-1^ immobilized the un-injected limb, indicating that systemic drug distribution occurred after local perineural injection. Peri-sciatic injection of neoSTX also resulted in potentially lethal events such as grasping for breath and apnea, and the LD_50_ was 4.9 μg/kg for sciatic perineural injection (Templin et al., 2015). A phase 1 study of neoSTX-Bup with or without epinephrine for cutaneous anesthesia revealed prolonged anesthesia with tolerable side effects such as dose-dependent perioral numbness and tingling in human volunteers (Lobo et al., 2015). Compared with neoSTX-Bup, QXOH-LB may have some advantages in term of systemic toxicity. As demonstrated in a pharmacodynamic study using rat sciatic nerve block models focusing on the efficacy and toxicity of multiple formulations of QX314 plus bupivacaine combination, the optimal formulation was 0.9% (25 mM) QX314 with 0.5% (15 mM) bupivacaine, which established approximately 10 h of non-selective sciatic nerve block with moderate local tissue inflammations (Yin et al., 2017). Unfortunately, combinations with QX314 ≥ 25 mM induce severe tissue inflammations. It seems that the QXOH and LB combination is probably superior to QX314 plus bupivacaine in terms of both anesthetic potency and adverse effects.

Local anesthetic effect is produced blocking the generation and propagation of action potentials by occupying intracellular sodium channels of axon after using by local anesthetics (Narahashi et al., 1970; Fink, 1989; Butterworth and Strichartz, 1990). Depended on the chemical structure, QXOH is permanently charged so that it was difficult to permeate the membrane passively. On the other hand, inner-cellular QXOH cannot expel from neuron easily. Therefore, the mechanism of the QXOH-induced long-acting effect might be related to its intracellular accumulation with slow leakage. It is currently clear that bupivacaine improves the cellular uptake of QX314 through TRP sub-family channels, such as TRPV1 and TRPA1, which are predominantly expressed in peripheral nociceptive neurons. QX-314 could also enter neurons through non-TRP pathways, because a selectively extended blockade of action potentials in C-fibers presented in neurons from TRPA1-knockout, TRPV1/TRPA1-double knockout, and wild-type mice (Brenneis et al., 2014). Considering the structural similarity between QX314 and QXOH, as well as that between LB and bupivacaine, we presumed that QXOH-LB might have the same mechanisms of action as QX314 plus bupivacaine. Because this study did not focus on the action mechanisms of QXOH-LB, the proofs for cellular entering pathways are not available. However, the characteristics of the nerve block induced by QXOH-LB were generally similar to those of the QX314 plus bupivacaine combination, which were prolonged, sensory-predominant nerve block. It has been proven that QXOH alone at 35 mM cannot produce sensory-selective nerve block (Zhang et al., 2017), and LB at 10 mM (approximately 0.3%) is not capable of producing a sensory-preferable nerve blockade in clinical settings. The co-application of these investigated drugs displayed a sensory-predominant nerve block, which at least suggested that some thermal nociceptive-expressed pathways might be involved.

This study has some noteworthy limitations and, therefore, the results should be interpreted with caution. First, we used only noxious heat to represent a nociceptive stimulus. There might be differences in the inhibition and recovery process between thermal sensation and that of mechanical or chemical sensations. Second, although QXOH-LB did not produce clinically important toxicities, further studies focusing on long-term and subtle tissue injury should be performed before considering the use of QXOH-LB for perineural applications.

Regardless of the above limitations, it was evident that QXOH in combination with LB induced long-lasting infiltration anesthesia as well as sciatic nerve block without clinical importantly side effects.

## Conclusion

In this study, we demonstrated that the fixed-dose combination, QXOH-LB, produced cutaneous anesthesia which was 2-fold greater than that produced by QXOH or LB alone, and elicited sciatic nerve block with a potency that was 5- and 3-fold that of LB and QXOH, respectively. Local tissue inflammation by QXOH and QXOH-LB was mild, similar to that induced by LB. The I.V. toxic doses of QXOH-LB were several-fold that of local application. This fixed-dose combination led to an antagonistic interaction between QXOH and LB in terms of systemic toxicity. These results suggested that QXOH-LB induced a long-lasting local anesthesia, likely, avoiding clinically important local and systemic toxicities. All these findings increase the potential for the clinical use of QXOH/LB. In the further experiments, we will focus on evaluating the effects of respiratory, cardiovascular and central nervous system by QXOH/LB in rats and beagle dogs according to Good Laboratory Practice (GLP) standard.

## Author Contributions

QY, YZ, WZ, and TZ conceived and designed the project. QY, YZ, RL, DG, BK, JY, and LT performed the experiments. QY, YZ, and WZ analyzed the data. QY and YZ wrote the manuscript. WZ and TZ revised the manuscript.

## Conflict of Interest Statement

The authors declare that the research was conducted in the absence of any commercial or financial relationships that could be construed as a potential conflict of interest.
